# Assessment of compliance to packaging and labeling regulatory requirements of locally manufactured alcohol-based hand sanitizers marketed in Addis Ababa, Ethiopia

**DOI:** 10.1186/s40545-022-00456-6

**Published:** 2022-10-10

**Authors:** Tesfa Marew Wallelign, Muluken Nigatu Selam, Gebremariam Birhanu Wondie, Bruck Messele Habte

**Affiliations:** 1 Department of Pharmaceutics and Social Pharmacy, School of Pharmacy, College of Health Sciences, Addis Ababa University, P.O. Box: 9086, Addis Ababa, Ethiopia; 2 Department of Pharmacology, Faculty of Health Sciences, University of the Free State, Bloemfontein, South Africa

**Keywords:** Hand sanitizers, Labeling requirements, Packaging requirements, Regulatory compliance

## Abstract

**Background:**

Following the emergence of the global Coronavirus Disease-2019 (COVID-19) pandemic, alcohol-based hand sanitizers (ABHS) have been extensively used as one of the effective methods of preventing its transmission. The products are dispensed over the counter and used by the general population. Growing concerns have been reported, however, regarding the quality, efficacy and compliance to regulatory requirements calling for objective evidence that can facilitate proactive regulatory measures.

**Objectives:**

The study aimed at assessing the level of compliance to packaging and labeling regulatory requirements of selected locally manufactured ABHS products marketed in Addis Ababa, Ethiopia.

**Methods:**

A cross-sectional study design was employed to randomly collect 25 locally manufactured ABHS products from retail outlets located in Addis Ababa. The manufacturers were grouped under four categories considering their experience in manufacturing, resources and technical capacities. The collected samples were evaluated for compliance to packaging and labeling information regulatory requirements and the results subjected to descriptive analysis.

**Results:**

Majority of the products were found to meet most of the packaging, general product description and manufacturer-related information requirements. However, concerning gaps were observed in storage, precaution and warning-related labeling information requirements. The overall compliance of the selected products (to a total of 29 requirements under 5 categories) was 56.9%. The highest level of compliance was for general product information requirements (80.2%) followed by packaging and manufacturer-related requirements accounting for 76.8% and 75.0%, respectively. Low level of compliance was observed for storage condition and precautions (10.2% and 42.4%, respectively). Better overall compliance to packaging and labeling requirements (62.9%) were observed by large pharmaceutical and cosmetics manufacturers, while the lowest compliance level was recorded for medium level pharmaceuticals and cosmetics manufacturers.

**Conclusions:**

Even though most of the selected products were able to comply with the majority of packaging, product description and manufacturer-related requirements, gaps were observed in essential labeling information requirements. Considering the extensive use of ABHS products among diverse population groups and the potential risks associated with inappropriate use of the products, improving regulatory law enforcement practices, strengthening continuing education of manufacturing personnel and raising public awareness is very timely.

**Supplementary Information:**

The online version contains supplementary material available at 10.1186/s40545-022-00456-6.

## Background

Ethyl alcohol-based preparations (70% Ethanol) have been extensively used for infection prevention in healthcare facilities and communities world-wide [1–3]. Following the emergence of the Coronavirus Disease-2019 (COVID-19) pandemic, hand hygiene has become the cornerstone of good infection prevention and control practices as it is simple to apply, affordable and demonstrated effectiveness in preventing community-based and healthcare-associated infections including COVID-19 [2, 4, 5]. *“Clean hands can protect you from serious infections while you are in a healthcare facility”* is a widely recognized motto and healthcare facilities have been implementing various strategies to contain the spread of communicable diseases. The use of Alcohol-Based Hand Sanitizers (ABHS) has been extensive, particularly when access to hand soap and clean water is limited [2, 6] and to keep the skin hydrated which otherwise can lead to cracked skin due to frequent hand washing [7]. The World Health Organization (WHO) strongly recommended the use of ABHS as the “gold standard” for hand disinfection in healthcare facilities and the community because of broad spectrum antimicrobial activity on various strains including Severe Acute Respiratory Syndrome Coronavirus 2 (SARS-CoV-2), easy availability at the point of care, better safety profile and general acceptability to users [8, 9]. Ethanol concentration of 80% and ≥ 60% are recommended by WHO and Center for Disease Control and Prevention (CDC), respectively, for the effective use of ABHS [4, 10].

Along with the increasing demand and increasing number of hand sanitizer manufacturers, quality assurance and regulatory functions have become complicated. It is an absolute fact that the effectiveness of the products is highly dependent on quality and proper use. In Ethiopia, different companies including large- and small-scale pharmaceutical manufacturers, healthcare supplies manufacturers, cosmetics manufacturers, hospitals and training and research centers are engaged in the production and marketing of ABHS to respond to the dire demand for the pandemic or to get benefit from the lucrative market.

In Ethiopia, hand sanitizers are regulated as over-the-counter (OTC) products [11]. The products are expected to meet the minimum quality, safety and efficacy requirements set by the regulatory body to ensure safe and informed use. Assurance of the compliance of product packaging and labeling information to regulatory requirements is equally important to the quality and safety of the product. The products are required by law to meet minimum packaging and labeling information regulatory requirements which may result in compromised outcome or health risks, if not complied to. The diverse packaging type and labeling information of such products in Ethiopia have been raised as a concern by the general public, health professionals and regulatory experts. Studies in other countries also revealed wide range of non-conformities including incomplete or missing label information, absence of ingredient lists, missing cautionary warnings, inclusion of unspecified colorants and fragrances, and formulating with suboptimal amount of active ingredients [12–14]. Because of lack of proper understanding on the impact of inappropriate packaging and/or labeling, or due to business orientation by manufacturers and supply chain actors, the problem has become pronounced calling for objective evidences which can be used for timely regulatory measures.

Recognizing the concerns and the need for objective evidences, the present study aimed to assess the compliance of packaging and labeling information to regulatory requirements of locally manufactured ABHS products marketed in Addis Ababa, Ethiopia.

## Methods

### Study area

Addis Ababa is the political and commercial capital of Ethiopia with a population of over 5 million accounting for about 25% of the country’s urban population [15]. The city is administratively divided into eleven sub cities and 116 Woredas. Addis Ababa is one of the fastest growing cities in Africa. The city is a diplomatic center for Africa and hosts a number of international organizations, such as the headquarters of African Union and the United Nations Economic Commission for Africa, among others [15]. The Ethiopia Airlines is also Africa’s largest fleet operating to different parts of the world accommodating several direct and transit flights and serving millions of the regional and international diplomatic and business communities. Because of the large market and access to facilities, pharmaceuticals and cosmetics manufacturing facilities, and distribution actors are largely concentrated in and around Addis Ababa.

### Study design

A cross-sectional study design was employed to collect locally manufactured ABHS products made available to the community in Addis Ababa, Ethiopia in October and November 2021.

### Source and study population

The source population was ABHS manufactured by local manufacturers and marketed to the community in Addis Ababa. Those products fulfilling the following eligibility criteria comprised the study population.

### Eligibility criteria

The ABHS that contained ethanol as an active ingredient, were in solution form, manufactured by local manufacturers, labeled with information, having usable shelf-life, and registered by the Ethiopian Food and Drug Authority (EFDA) were included in the study.

### Sample size and sampling techniques

According to sources from EFDA, there were 161 hand sanitizer manufactures licensed by the authority nationwide, of which 124 were from Addis Ababa and its outskirts. The sanitizer manufacturers have different capacities and experiences in pharmaceuticals or cosmetics manufacturing. For the effective promotion of public health, production and marketing of pharmaceuticals are stringently regulated. In addition to regular inspection and law enforcement by regulators, the authors believe that capacity and experience of manufacturers can influence quality systems implementation, product quality, marketing and sales practices, and level of compliance to regulatory requirements. With this basic understanding, and for better interpretation of findings, ABHS manufacturers from Addis Ababa and its environs were broadly categorized into four: (i) large-scale pharmaceutical and cosmetics/chemicals manufacturers (17); (ii) medium level cosmetics and chemical manufacturers (31); (iii) small scale pharmaceuticals and other healthcare supplies manufacturers (34); and (iv) other small firms established following the COVID-19 pandemic (42).

Among the 124 manufacturers, considering resource constraints and sample size representativeness, a total of 25 products (20% from each category of manufacturers) were included for the assessment of packaging and compliance to labeling regulatory requirements. Products from each of the four categories with reserves were randomly selected.

### Sample collection procedure

The sample collection was carried out by the first and second authors who purchased them from drug retail outlets and supermarkets in Addis Ababa until the required sample size was reached. Sample products that were in the package size ranges from 250 to 1000 ml were purchased from the retail outlets as found appropriate.

### Evaluation of packaging and labeling compliance of ABHS to regulatory requirements

Alcohol-based hand sanitizers are among the OTC products that are being widely and more frequently used by the general population with varied age groups, occupation and educational background. The presence of clear and adequate information on the primary packaging container is very essential to promote rational and economic use of the products and to avoid preventable health risks. In its temporary manufacturing, import and distribution licensing directive for COVID-19 inputs, EFDA established packaging requirements (tightly sealed, suitable for use, child resistant and can protect the product) and labeling requirements (product information, precautions and warnings, and manufacturer information) for ABHS products. The authority has also indicated that US Food and Drug Administration (USFDA) approval certificate and WHO prequalification certificate are recognized for temporary licensing. Accordingly, an assessment checklist extracted from EFDA temporary licensing directive, USFDA Temporary Authorization Policy and USP-NF and FCC Standards for ABHS [16–18] and contextualized to the local scenario was used.

The selected sample products were then evaluated by the first author in collaboration with the second author for their compliance to the four category of regulatory requirements: (i) primary packaging type and integrity (tightly sealed, suitable for use, child resistant and can protect the product); (ii) general product descriptions (physical characteristics, formulation composition and storage conditions); precautions and warnings (directions for use, “do not use” advices and recommendations in cases of accidental exposure); and (iv) manufacturer’s detail and contact information.

### Data analysis and interpretation

Data were properly collected, coded, analyzed and presented using descriptive statistics, and results were presented using tables and figure as found appropriate.

### Data quality assurance

The data collection and analysis were carried out by the first and second authors who have theoretical and practical expertise in the area. Pictures of the products were taken and confidentially kept. Information recording was double-checked by different investigators during the time of recording.

### Ethical consideration

Before starting the research work, ethical clearance was obtained from the Ethical Review Board of the School of Pharmacy, College of Health Sciences, Addis Ababa University (ERB/SOP/307/13/2021). All the information collected from the study were coded and maintained confidential.

## Results

Following the global COVID-19 pandemic, several organizations ranging from established pharmaceuticals and cosmetics manufacturers to newly established firms started manufacturing ABHS to respond to the dire demand with the sense of social corporate responsibility or business interest. Level of experience in the area, resource and technical capacity and the level of regulatory enforcement in light of the unforeseen pandemic can influence the degree of compliance to regulatory requirements by the companies. With this understanding, the authors categorized the companies into four following in-depth discussion about the respective companies.

The list and overall description of the selected products is presented in Table 1. Based on regulatory and safety requirements, the selected products under the respective categories were evaluated for required classes of parameters including: (i) primary packaging type and integrity; (ii) general product information and storage conditions; (iii) precautions and “do not use advices”; and (iv) manufacturers’ information.

**Table 1:** Description of locally manufactured hand sanitizer products marketed in Addis Ababa, Ethiopia, 2021

No	Product code	Product description				
		Expiry date (month/year)	Pack size	Colorant	Fragrance	Cap type
1	LPC101	10//2022	1000 ml	No	Yes	Screw
2	LPC102	04//2024	1000 ml	No	No	Screw
3	LPC103	04/2022	1000 ml	No	No	Screw
4	LPC104	05/2023	500 ml	No	No	Spray
5	MPC201	Not indicated	250 ml	No	No	Spray
6	MPC202	Not indicated	1000 ml	No	Yes	Screw
7	MPC203	04/2024	1000 ml	No	No	Screw
8	MPC204	04/2023	1000 ml	No	No	Screw
9	MPC205	Not indicated	1000 ml	No	No	Screw
10	MPC206	Not indicated	500 ml	No	No	Spray
11	SPC301	12/2023	1000 ml	No	No	Screw
12	SPC302	05/2023	1000 ml	Yes	No	Screw
13	SPC303	Not indicated	1000 ml	Yes	No	Screw
14	SPC304	04/2023	1000 ml	Yes	No	Screw
15	SPC305	03/2022	500 ml	No	No	Screw
16	SPC306	06/2023	1000 ml	Yes	Yes	Screw
17	SPC307	Not indicated	500 ml	Yes	Yes	Spray
18	SSC401	15/2022	500 ml	No	No	Pull–push
19	SSC402	05/2022	1000 ml	No	No	Screw
20	SSC403	06/2023	1000 ml	No	No	Screw
21	SSC404	12/2022	1000 ml	No	No	Screw
22	SSC405	01/2022	500 ml	No	No	Screw
23	SSC406	11/2022	1000 ml	No	No	Screw
24	SSC407	03/2022	500 ml	No	No	Spray
25	SSC408	Not indicated	250 ml	Yes	No	Flip-top-drop

*LPC* larger pharmaceutical and cosmetics manufacturing companies, *MPC* medium size pharmaceutical and cosmetics manufacturing companies, *SPC* small scale pharmaceuticals and chemicals manufacturing companies, *SSC* small scale Covid-19 sanitizer manufacturing companies

### Level of compliance to packaging requirements and general product characteristics

All of the 25 products were contained in Low Density Polyethylene Terephthalate (LDPET) plastic bottles. No leakage was observed and the tamper indicator strip seal was intact in all of the selected products. Except for two products from category 3 (small-scale pharmaceuticals and cosmetics manufacturing firms), the containers were simple transparent plastics. All the products were found capped with Polyethylene Terephthalate (PET) plastic caps of different design and colors. Eighteen products (72%) had screw caps followed by spray caps (5), push–pull (1), and flip-top-drop type (1).

All the procured products were solution types. Majority of the products were prepared as colorless and odorless solutions. Six products (24%) had different types of colorants, while only three products (12%) contained fragrances as specified from the labeling information. Surprisingly, five of the seven products (71.4%) from category 3 firms had colorants. However, the type and concentration of the colorants and fragrances in products were not described in the product labeling information.

### Formulation and product information-related requirements

**Fig. 1:**
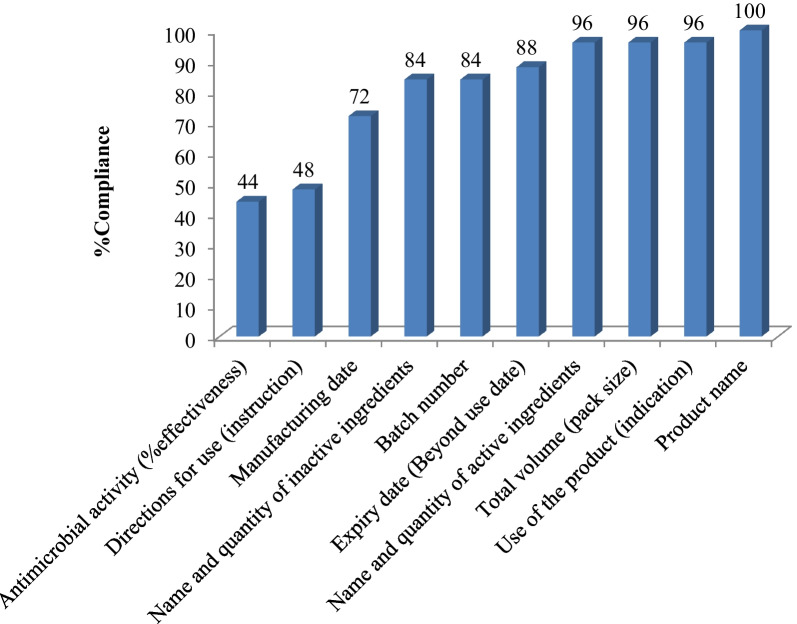
Overall compliance level of selected ABHS products to formulation and product information requirements (*N* = 25)

The selected products were examined for the presence of clear and legible product information on the package label as stipulated in the temporary licensing and registration guideline formulated by the EFDA. The products were purchased in their primary container and no package insert (patient information sheet) was provided during the transaction. The product name and total volume was clearly described in almost all the selected products (100% and 96%, respectively). The names and quantities of active ingredients and formulation additives were also indicated in most of the product labels (96% and 84%, respectively). Failure to include instruction for use in the product package labels was observed in more than half of the products (52%) and no explanation was provided about instruction for use while purchasing the products. On the other hand, majority of the manufacturers, except for those under Category 1 (large pharmaceuticals and cosmetics manufacturers) indicated antimicrobial effectiveness of their products by specifically describing it as “kills 99.9% of germs” (details presented as Additional file 1). The detailed number of products meeting the labeling requirements under the different categories is depicted in Fig. 1.

### Precautions and warning-related information

All the four manufacturers from category 1 included most of the precautionary information except for some manufacturers who failed to indicate to avoid contact and seek medical help in cases of exposure. However, majority of them failed to include important “do not use advises” such as: (i) do not use on open skin/wounds; (ii) do not use in children less than 2 months; and (iii) supervise children under 6 years. Similar trends were observed for manufacturers from the other categories in describing precautions, while all the companies in the remaining three categories did not include the important “do not use” advises in the labeling information as can be observed in Table 2. The average level of compliance to warning/precautions and do not use related labeling requirements were 59.2% and 2.7%, respectively, while the overall average level of compliance to requirements under this category was 38%.

**Table 2:** Number and percentage of product labels complying to precaution and warning information requirements among the selected ABHS manufacturers, Addis Ababa, 2021

Labeling requirements	Category 1 (4)	Category 2 (6)	Category 3 (7)	Category 4 (8)	Total (25)
*Warnings/precautions*					
For external use only	4 (100)	4 (66.7)	3(42.9)	7(87.5)	18(72.0)
Keep out of reach of children	4(100)	3(50.0)	6(85.7)	6(75.0)	19(76.0)
Flammable: Keep away from flame and heat	4(100)	4(66.7)	5(71.4)	5(62.5)	18(72.0)
Avoid contact with eyes, ears, mouth; rinse with water	3(75)	2(33.3)	4(57.1)	5(62.5)	14(56.0)
If swallowed, get medical help right away	2(50)	2(33.3)	1(14.3)	0(0)	5(20.0)
*Do not use advises*					
Do not use on open skin/wounds	1 (25)	0(0)	0(0)	0(0)	1(4.0)
Do not use in children less than 2 months	1(25)	0(0)	0(0)	0(0)	1(4.0)
Supervise children under 6 years	0(0)	0(0)	0(0)	0(0)	0(0)

### Manufacturer’s information

Complete information and contact address of the manufacturer is a legal requirement for production and marketing of regulated products including hand sanitizers. From the study, all the 25 products were found to contain the name and contact address of the manufacturing company, including country of origin. It was also found that 22 products contain unique product names (brands) in addition to the conventional product name, i.e., “alcohol-based hand sanitizer” or “hand sanitizer”. The Ethiopian Standards Agency plays a role in developing national standards for local products including the ABHS. However, none of the products contain the Ethiopian Standards Mark; an indicator that confirms the marketed product meets the minimum standards set for hand sanitizer products. This particular requirement is not mandatory and the EFDA has not described it as a regulatory requirement.

### Overall compliance level of selected products to packaging and labeling requirements

As observed in Table 3, the overall compliance of the selected ABHS products (a total of 29 requirements under 5 categories) was 56.9%. The highest level of compliance was for general product information requirements (80.2%) followed by packaging and manufacturer-related requirements accounting for 76.8% and 75.0%, respectively. Concerning low level of compliance, it was observed for storage condition and precautions/warnings (10.2% and 42.4%, respectively).

**Table 3:** Average and overall compliance to packaging and labeling requirements of selected ABHS manufacturers by company category, Addis Ababa, 2021

Category of requirements	Number of requirements	Categories of manufacturers and percentage compliance to requirements				
		Category 1	Category 2	Category 3	Category 4	Average
Packaging type and integrity	4	75	75	82.2	75	76.8
General product information	10	80	68.3	88.6	83.8	80.2
Precautions and “do not use” advises	8	59.4	31.3	42.9	35.9	42.4
Storage conditions	3	25	11.1	4.8	0	10.2
Manufacturer information	4	75	75	75	75	75.0
Total/average	29	62.9	52.1	58.7	53.9	56.9

With regard to the level of compliance by category of companies, better overall compliance to packaging and labeling requirements were observed by Category 1 companies (large pharmaceutical and cosmetics manufacturers, 62.9%), while the lowest compliance level was recorded by Category 2 companies (established medium level pharmaceuticals and cosmetics manufacturers, with overall compliance level of 52.1%). The detailed compliance level to specific category of packaging and labeling requirements and overall compliance by the different groups are presented in Table 3.

## Discussion

Following the COVID-19 pandemic, the use of ABHS has been praised as one of the effective preventive measures, particularly when access to soap and water is limited [19, 20]. The efficacy of ABHS, however, depends on, among the others, on the quantity applied, the technique used, and the consistency of use that emanate from the appropriate labeling information indicated on the products’ container [21]. On the other hand, quality and safety issues related to the extensive and inappropriate use of ABHs have become persistent among professionals, researchers, regulators and the general public [22, 23].

As described above, ABHS are dispensed under OTC drug classes and often made available in pharmacies, cosmetics shops, super markets, ordinary shops, and even on open street markets in some instances. In addition to potential health risks, inappropriate handling and use of these products may lead to fire and explosion hazards. In consideration of this, strict compliance to labeling information requirements is expected from manufacturers. All precautions, warnings and “do not use” advices need to be clearly visible on the primary packaging labeling information.

Proper packaging with clear labeling information can enhance rational use of ABHs and also reduce potential risks associated with inappropriate use. Because of the extensive use among population of diverse age and level of awareness, the products are expected to strictly comply with packaging and labeling regulatory requirements. This study showed that most of the products included could meet most of packaging and general product information-related requirements (76.8% and 80.2%, respectively). However, almost all primary packaging containers could not protect product against exposure to light which may increase the chance of product deterioration and progressively reduce effectiveness. It was also observed that most of the selected products are free off colorants and fragrances and were based on the WHO recommended formulation consideration. However, a few of the manufacturers that used colorants and/or fragrances failed to clearly describe the type and concentration of the colorant and fragrant used; which may be difficult to prevent avoidable skin reactions and other health risks.

With regard to information on microbial effectiveness of ABHS, it is becoming a common practice for most products circulating in the market to indicate the level of effectiveness, usually as 99.9%, in their labeling information, though it is not among the regulatory requirements for hand sanitizers labeling information. This effectiveness information may be included to merely attract markets, although such claims needed to be assessed and verified by the manufacturers and/or the regulatory before marketing [24]. In the current study, none of the manufacturers under Category I mentioned this information in their labels compared to more than half of the small and medium level manufacturers (Category II–IV) who cited the antimicrobial effectiveness information in their labels. The manufacturers under Category I did not mention this information on their labels possibly be due to their awareness and experience with the regulatory requirements, in that only information that are verified or proven by experiments need to be disseminated and protecting the consumers from the false sense of security [24].

Effective and safe use of ABHS depends on the amount applied at a time, application time, following standard technique and the drying time. In this regard, user friendly packaging and clear labeling information play critical role in promoting effective utilization, and prevention of avoidable health risks [25, 26]. In the present study, only 47.6% of the 25 products included in the study presented instruction for use. Better compliance was observed among companies in Category 3 (57.1%), while the lowest compliance was recorded among manufacturers in Category 2 (33.3%). Alarmingly low levels of compliance were observed for requirements related to “precautions and do not use advises” and storage conditions (42.4% and 10.2%, respectively). Similarly, inadequate labeling information in addressing direction for use, precautions and storage condition were reported by a study conducted in Nairobi, Kenya [13]. These regulatory requirements are very critical—compliance can enhance rational and effective product use, while incompliances may lead to safety and health risks. Even though majority of the product labels were found to include “keep out of the reach of children” information, almost all fail to warn supervising the use in children below the age of 6 and restriction of use for children less than 2 months. Hand sanitizer products are marketed using attractive packaging designs and also with colorants and fragrances which may result in over use and/or poisoning of young children have been reported [27, 28].

Alcohol-based hand sanitizers are very flammable, can often cause health risks upon accidental or intentional ingestion, and do have adverse effects when used on broken skin and exposure to the eyes [29, 30]. In this study, the overall levels of “keep away from fire and heat” and “avoid exposure to ears, eyes and mouth” compliance were 75.2% and 57.0%, respectively. The health and environment implications of missing such critical requirements need to be proactively communicated among regulatory experts, manufacturers, distributors, healthcare professionals and the public. User harms including severe burns have been reported because of inappropriate handling and use of hand sanitizer products [31]. Critical incidents in children that have used ABHSs have also been reported [27]. The use of ABHS that are adulterated with methanol can further result in potential toxicity when accidentally ingested particularly by children [28].

Requirements related to the details of product manufacturers were fulfilled by all. However, none of the product labels had the “Ethiopian Standards Mark” which has become mandatory requirement in different product categories, and can be considered as an indication for better compliance to quality and safety requirements. Products under Category 1 companies exhibited the maximum overall compliance to the packaging and labeling requirements as compared with products under other categories which might be due to the experience in production of medicinal products and their frequent communication with the regulatory body (EFDA) for product registration and marketing.

The lower level of compliance in some of the critical requirements by the manufacturers may be associated with lack of proper understanding of the implications by the different stakeholders, and the weak regulatory enforcement practices (in consideration of the increasing demand to respond to the pandemic). Proper labeling can assist in controlling the availability of falsified medicines in the circulating market [32]. On the other hand, the failure of manufacturers to comply with regulatory requirements of labeling for their medicinal products can lead to the suspension, registration cancellation or even product recall and civil lawsuit [33, 34]. It is also a common observation that ABHSs are marketed in regular shops and even on open street markets without proper counseling and associated product labeling information. It is thus evident that the problem is not limited to just the packaging and labeling aspect but also other aspects including the effectiveness of the products which can be considered a limitation of this study. This study was actually part of a larger study which has addressed the effectiveness of these products among other things. Such practices in any case call for timely interventions in law enforcement and public health education but also continual trainings to the manufacturing personnel on the different aspects.

## Conclusions

Majority of the selected products were found to meet most of the packaging, general product description and manufacturer-related requirements. The overall compliance of the selected products was 56.9%. Better level of compliance was observed in products general product information requirements and manufacturer-related requirements. Major gaps were observed in essential labeling information requirements such as instruction for use, precautions/warnings, and do not used advices. Considering the extensive use among diverse population groups, and the potential health risks associated with inappropriate use of the products, improving regulatory law enforcement practices, strengthening continuing education of manufacturing personnel and raising public awareness about the rational use of the products is very timely.

## Supplementary Information

**Additional file 1.** Compliance level of packaging and labeling information requirements of selected ABHS products by company Category.

## Data Availability

The detailed analysis used for the study is included as supplementary data. The datasets used and/or analyzed during the current study on the other hand are available from the corresponding author on reasonable request.

## Abbreviations

ABHS: Alcohol-based hand sanitizer
COVID-19: Coronavirus Disease-2019
EFDA: Ethiopian Food and Drug Authority
OTC: Over-the-counter
USFDA: US Food and Drug Administration
WHO: World Health Organization
