# Supplementary material for: Assessment of compliance to packaging and labeling regulatory requirements of locally manufactured alcohol-based hand sanitizers marketed in Addis Ababa, Ethiopia
Source: J Pharm Policy Pract. 2022 Oct 10;15:60. doi: 10.1186/s40545-022-00456-6 (PMC9550303; doi:10.1186/s40545-022-00456-6)
Supplement: Supplementary file 1 — Additional file 1. Compliance level of packaging and labeling information requirements of selected ABHS products by company Category. [file 40545_2022_456_MOESM1_ESM.docx]

**Supplementary Information**

**Annex I: Compliance level of packaging and labeling information requirements of selected EBHS products by company Category**

**Category I**

| **Requirement category** | **Detailed parameter** | **Companies** | | | | |
| --- | --- | --- | --- | --- | --- | --- |
|  |  | LPC101 | LPC102 | LPC103 | LPC104 | Avg. %Compliance |
| Packaging type and integrity | Type of container (plastic) | ✓ | ✓ | ✓ | ✓ | 100.0 |
|  | Tightly sealed (no leakage) | ✓ | ✓ | ✓ | ✓ | 100.0 |
|  | Tamper resistant seal | ✓ | ✓ | ✓ | ✓ | 100.0 |
|  | Protect from light exposure | - | - | - | - | 0.0 |
|  | **Average compliance** | | | | | **75.0** |
| General product information | Product name | ✓ | ✓ | ✓ | ✓ | 100.0 |
|  | Name and quantity of active ingredients | ✓ | - | ✓ | ✓ | 75.0 |
|  | Name and quantity of inactive ingredients | ✓ | - | ✓ | ✓ | 75.0 |
|  | Total volume (pack size) | ✓ | ✓ | ✓ | ✓ | 100.0 |
|  | Use of the product (indication) | ✓ | ✓ | ✓ | ✓ | 100.0 |
|  | Directions for use (instruction) | - | - | ✓ | ✓ | 50.0 |
|  | Antimicrobial activity (%effectiveness) | - | - | - | - | 0.0 |
|  | Batch number | ✓ | ✓ | ✓ | ✓ | 100.0 |
|  | Manufacturing date | ✓ | ✓ | ✓ | ✓ | 100.0 |
|  | Expiry date (Beyond use date) | ✓ | ✓ | ✓ | ✓ | 100.0 |
|  | **Average compliance** | | | | | **80.0** |
| Precautions and  “do not use” advises | For external use only | ✓ | ✓ | ✓ | ✓ | 100.0 |
|  | Keep out of reach of children | ✓ | ✓ | ✓ | ✓ | 100.0 |
|  | Flammable: Keep away from flame and heat | ✓ | ✓ | ✓ | ✓ | 100.0 |
|  | Avoid contact with eyes, ears, mouth and rinse with water | ✓ | ✓ | ✓ | - | 75.0 |
|  | If swallowed, get medical help right away | - | ✓ | - | ✓ | 50.0 |
|  | Do not use on open skin/wounds | - | - | - | ✓ | 25.0 |
|  | Do not use in children less than 2 months | - | - | - | ✓ | 25.0 |
|  | Supervise children under 6 years | - | - | - | - | 0.0 |
|  | **Average compliance** | | | | | **59.4** |
| Storage conditions | Store between 15-30°C | - | ✓ | - | ✓ | 50.0 |
|  | Avoid freezing/ excessive heat above 40°C | - | - | ✓ | - | 25.0 |
|  | Protect from light | - | - | - | - | 0.0 |
|  | **Average compliance** | | | | | **25.0** |
| Manufacturer information | Name of the manufacturer | ✓ | ✓ | ✓ | ✓ | 100.0 |
|  | Contact address | ✓ | ✓ | ✓ | ✓ | 100.0 |
|  | Country of origin | ✓ | ✓ | ✓ | ✓ | 100.0 |
|  | Ethiopian standards mark | - | - | - | - | 0.0 |
|  | **Overall average compliance** | | | | | **75.0** |

**Category 2**

| **Requirement category** | **Detailed parameter** | **Companies** | | | | | | |
| --- | --- | --- | --- | --- | --- | --- | --- | --- |
|  |  | MPC201 | MPC202 | MPC203 | MPC204 | MPC205 | MPC206 | Avr. %Compliance |
| Packaging type and integrity | Type of container (plastic) | ✓ | ✓ | ✓ | ✓ | ✓ | ✓ | 100.0 |
|  | Tightly sealed (no leakage) | ✓ | ✓ | ✓ | ✓ | ✓ | ✓ | 100.0 |
|  | Tamper resistant seal | ✓ | ✓ | ✓ | ✓ | ✓ | ✓ | 100.0 |
|  | Protect from light exposure | - | - | - | - | - | - | 0.0 |
|  | **Average compliance** | | | | | | | **75.0** |
| General product information | Product name | ✓ | ✓ | ✓ | ✓ | ✓ | ✓ | 100.0 |
|  | Name and quantity of active ingredients | ✓ | ✓ | ✓ | ✓ | ✓ | ✓ | 100.0 |
|  | Name and quantity of inactive ingredients | - | - | ✓ | ✓ | ✓ | ✓ | 66.7 |
|  | Total volume (pack size) | ✓ | ✓ | ✓ | ✓ | ✓ | ✓ | 100.0 |
|  | Use of the product (indication) | ✓ | ✓ | ✓ | ✓ | ✓ | ✓ | 100.0 |
|  | Directions for use (instruction) | - | - | - | ✓ | ✓ | - | 33.3 |
|  | Antimicrobial activity (%effectiveness) | ✓ | ✓ | ✓ | - | - | - | 50.0 |
|  | Batch number | - | - | ✓ | ✓ | - | ✓ | 50.0 |
|  | Manufacturing date | - | - | ✓ | ✓ | - | - | 33.3 |
|  | Expiry date (Beyond use date) | - | - | ✓ | ✓ | - | ✓ | 50.0 |
|  | **Average compliance** | | | | | | | **68.3** |
| Precautions and  “do not use” advises | For external use only | - | - | ✓ | ✓ | ✓ | ✓ | 66.7 |
|  | Keep out of reach of children | - | - | - | ✓ | ✓ | ✓ | 50.0 |
|  | Flammable: Keep away from flame and heat | - | - | ✓ | ✓ | ✓ | ✓ | 66.7 |
|  | Avoid contact with eyes, ears, mouth and rinse with water | - | - | ✓ | - | ✓ | - | 33.3 |
|  | If swallowed, get medical help right away | - | - | ✓ | - | ✓ | - | 33.3 |
|  | Do not use on open skin/wounds | - | - | - | - | - | - | 0.0 |
|  | Do not use in children less than 2 months | - | - | - | - | - | - | 0.0 |
|  | Supervise children under 6 years | - | - | - | - | - | - | 0.0 |
|  | **Overall average compliance level** | | | | | | | **31.3** |
| Storage conditions | Store between 15-30°C | - | - | - | - | ✓ | - | 16.7 |
|  | Avoid freezing/ excessive heat above 40°C | - | - | - | - | ✓ | - | 16.7 |
|  | Protect from light | - | - | - | - | - | - | 0.0 |
|  | **Average compliance** | | | | | | | **11.1** |
| Manufacturer information | Name of the manufacturer | ✓ | ✓ | ✓ | ✓ | ✓ | ✓ | 100.0 |
|  | Contact address | ✓ | ✓ | ✓ | ✓ | ✓ | ✓ | 100.0 |
|  | Country of origin | ✓ | ✓ | ✓ | ✓ | ✓ | ✓ | 100.0 |
|  | Ethiopian standards mark | - | - | - | - | - | - | 0.0 |
|  | **Overall average compliance** | | | | | | | **75.0** |

**Category 3**

| **Requirement category** | **Detailed parameter** | **Companies** | | | | | | | |
| --- | --- | --- | --- | --- | --- | --- | --- | --- | --- |
|  |  | SPC301 | SPC302 | SPC303 | SPC304 | SPC305 | SPC306 | SPC307 | Avr. %Compliance |
| Packaging type and integrity | Type of container (plastic) | ✓ | ✓ | ✓ | ✓ | ✓ | ✓ | ✓ | 100.0 |
|  | Tightly sealed (no leakage) | ✓ | ✓ | ✓ | ✓ | ✓ | ✓ | ✓ | 100.0 |
|  | Tamper resistant seal | ✓ | ✓ | ✓ | ✓ | ✓ | ✓ | ✓ | 100.0 |
|  | Protect from light exposure | - | - | ✓ | - | - | ✓ | - | 28.6 |
|  | **Average compliance** | | | | | | | | **82.2** |
| General product information | Product name | ✓ | ✓ | ✓ | ✓ | ✓ | ✓ | ✓ | 100.0 |
|  | Name and quantity of active ingredients | ✓ | ✓ | ✓ | ✓ | ✓ | ✓ | ✓ | 100.0 |
|  | Name and quantity of inactive ingredients | ✓ | ✓ | ✓ | ✓ | ✓ | ✓ | ✓ | 100.0 |
|  | Total volume (pack size) | ✓ | ✓ | ✓ | ✓ | ✓ | ✓ | ✓ | 100.0 |
|  | Use of the product (indication) | ✓ | ✓ | ✓ | ✓ | ✓ | ✓ | ✓ | 100.0 |
|  | Directions for use (instruction) | ✓ | - | - | ✓ | ✓ | ✓ | - | 57.1 |
|  | Antimicrobial activity (%effectiveness) | ✓ | ✓ | - | - | - | ✓ | ✓ | 57.1 |
|  | Batch number | ✓ | ✓ | ✓ | ✓ | ✓ | ✓ | ✓ | 100.0 |
|  | Manufacturing date | ✓ | - | ✓ | ✓ | ✓ | - | ✓ | 71.4 |
|  | Expiry date (Beyond use date) | ✓ | ✓ | ✓ | ✓ | ✓ | ✓ | ✓ | 100.0 |
|  | **Average compliance** | | | | | | | | **88.6** |
| Precautions and  “do not use” advises | For external use only | - | - | - | ✓ | ✓ | ✓ | - | 42.9 |
|  | Keep out of reach of children | ✓ | - | ✓ | ✓ | ✓ | ✓ | ✓ | 85.7 |
|  | Flammable: Keep away from flame and heat | ✓ | - | ✓ | ✓ | ✓ | ✓ | - | 71.4 |
|  | Avoid contact with eyes, ears, mouth and rinse with water | ✓ | - | ✓ | - | ✓ | ✓ | - | 57.1 |
|  | If swallowed, get medical help right away | - | - | ✓ | - | - | - | - | 14..3 |
|  | Do not use on open skin/wounds | - | - | - | - | - | - | - | 0.0 |
|  | Do not use in children less than 2 months | - | - | - | - | - | - | - | 0.0 |
|  | Supervise children under 6 years | - | - | - | - | - | - | ✓ | 14..3 |
|  | **Average compliance** | | | | | | | | **42.9** |
| Storage conditions | Store between 15-30°C | - | - | - | - | - | - | ✓ | 14..3 |
|  | Avoid freezing/ excessive heat above 40°C | - | - | - | - | - | - | - | 0.0 |
|  | Protect from light | - | - | - | - | - | - | - | 0.0 |
|  | **Average compliance** | | | | | | | | **4.8** |
| Manufacturer information | Name of the manufacturer | ✓ | ✓ | ✓ | ✓ | ✓ | ✓ | ✓ | 100.0 |
|  | Contact address | ✓ | ✓ | ✓ | ✓ | ✓ | ✓ | ✓ | 100.0 |
|  | Country of origin | ✓ | ✓ | ✓ | ✓ | ✓ | ✓ | ✓ | 100.0 |
|  | Ethiopian standards mark | - | - | - | - | - | - | - | 0.0 |
|  | **Overall average compliance** | | | | | | | | **75.0** |

**Category 4**

| **Requirement category** | **Detailed parameter** | **Companies** | | | | | | | | |
| --- | --- | --- | --- | --- | --- | --- | --- | --- | --- | --- |
|  |  | SSC401 | SSC402 | SSC403 | SSC404 | SSC405 | SSC406 | SSC407 | SSC408 | Avr. %Compliance |
| Packaging type and integrity | Type of container (plastic) | ✓ | ✓ | ✓ | ✓ | ✓ | ✓ | ✓ | ✓ | 100 |
|  | Tightly sealed (no leakage) | ✓ | ✓ | ✓ | ✓ | ✓ | ✓ | ✓ | ✓ | 100 |
|  | Tamper resistant seal | ✓ | ✓ | ✓ | ✓ | ✓ | ✓ | ✓ | ✓ | 100 |
|  | Protect from light exposure | - | - | - | - | - | - | - | - | 0 |
|  | **Average compliance** | | | | | | | | | **75.0** |
| General product information | Product name | ✓ | ✓ | ✓ | ✓ | ✓ | ✓ | ✓ | ✓ | 100 |
|  | Name and quantity of active ingredients | ✓ | ✓ | ✓ | ✓ | ✓ | ✓ | ✓ | ✓ | 100 |
|  | Name and quantity of inactive ingredients | ✓ | - | ✓ | ✓ | ✓ | ✓ | ✓ | ✓ | 87.5 |
|  | Total volume (pack size) | ✓ | - | ✓ | ✓ | ✓ | ✓ | ✓ | ✓ | 87.5 |
|  | Use of the product (indication) | ✓ | - | ✓ | ✓ | ✓ | ✓ | ✓ | ✓ | 87.5 |
|  | Directions for use (instruction) | - | - | - | ✓ | ✓ | ✓ | ✓ | - | 50 |
|  | Antimicrobial activity (%effectiveness) | - | ✓ | - | - | ✓ | ✓ | ✓ | - | 50 |
|  | Batch number | ✓ | ✓ | ✓ | ✓ | ✓ | ✓ | ✓ | - | 87.5 |
|  | Manufacturing date | ✓ | ✓ | ✓ | ✓ | ✓ | ✓ | ✓ | - | 87.5 |
|  | Expiry date (Beyond use date) | ✓ | ✓ | ✓ | ✓ | ✓ | ✓ | ✓ | ✓ | 100.0 |
|  | **Average compliance** | | | | | | | | | **83.8** |
| Precautions and  “do not use” advises | For external use only | ✓ | ✓ | ✓ | ✓ | ✓ | ✓ | ✓ | - | 87.5 |
|  | Keep out of reach of children | ✓ | ✓ | ✓ | ✓ | ✓ | - | ✓ | - | 75.0 |
|  | Flammable: Keep away from flame and heat | ✓ | ✓ | ✓ | ✓ | - | ✓ | - | - | 62.5 |
|  | Avoid contact with eyes, ears, mouth and rinse with water | ✓ | ✓ | - | ✓ | - | - | ✓ | ✓ | 62.5 |
|  | If swallowed, get medical help right away | - | - | - | - | - | - | - | - | 0.0 |
|  | Do not use on open skin/wounds | - | - | - | - | - | - | - | - | 0.0 |
|  | Do not use in children less than 2 months | - | - | - | - | - | - | - | - | 0.0 |
|  | Supervise children under 6 years | - | - | - | - | - | - | - | - | 0.0 |
|  | **Average compliance** | | | | | | | | | **35.9** |
| Storage conditions | Store between 15-30°C | - | - | - | - | - | - | - | - | 0.0 |
|  | Avoid freezing/ excessive heat above 40°C | - | - | - | - | - | - | - | - | 0.0 |
|  | Protect from light | - | - | - | - | - | - | - | - | 0.0 |
|  | **Average compliance** | | | | | | | | | **0.0** |
| Manufacturer information | Name of the manufacturer | ✓ | ✓ | ✓ | ✓ | ✓ | ✓ | ✓ | ✓ | 100.0 |
|  | Contact address | ✓ | ✓ | ✓ | ✓ | ✓ | ✓ | ✓ | ✓ | 100.0 |
|  | Country of origin | ✓ | ✓ | ✓ | ✓ | ✓ | ✓ | ✓ | ✓ | 100.0 |
|  | Ethiopian standards mark | - | - | - | - | - | - | - | - | 0.0 |
|  | **Average compliance** | | | | | | | | | **75.0** |
|  | **Overall average compliance** | | | | | | | | |  |

***Note: “****✓”refers to “yes” and “-” refers “no”.*
